# Animal Models for Investigating Osseointegration: An Overview of Implant Research over the Last Three Decades

**DOI:** 10.3390/jfb15040083

**Published:** 2024-03-27

**Authors:** Antonio Scarano, Ahmad G. A. Khater, Sergio Alexandre Gehrke, Francesco Inchingolo, Sergio Rexhep Tari

**Affiliations:** 1 Department of Innovative Technologies in Medicine and Dentistry, University of Chieti–Pescara, 66100 Chieti, Italy; sergiotari@yahoo.it; 2 Faculty of Oral and Dental Medicine, Egyptian Russian University (ERU), Badr City 11829, Egypt; ahmed.g.a.khater@gmail.com; 3 Health Affairs Directorate, Egyptian Ministry of Health and Population, Banisuif 62511, Egypt; 4 Department of Research, Bioface/PgO/UCAM, Montevideo 11100, Uruguay; sergio.gehrke@hotmail.com; 5 Department of Interdisciplinary Medicine, Section of Dental Medicine, University of Bari “Aldo Moro”, 70124 Bari, Italy; francesco.inchingolo@uniba.it

**Keywords:** ARRIVE guidelines, biomaterial, dental implants, in vivo studies, osseointegration, preclinical studies, regenerative medicine, translational research

## Abstract

Dental implants and bone augmentation are among dentistry’s most prevalent surgical treatments; hence, many dental implant surfaces and bone grafts have been researched to improve bone response. Such new materials were radiologically, histologically, and histomorphometrically evaluated on animals before being used on humans. As a result, several studies used animals to evaluate novel implant technologies, biocompatibility, surgical techniques, and osseointegration strategies, as preclinical research on animal models is essential to evaluate bioactive principles (on cells, compounds, and implants) that can act through multiple mechanisms and to predict animal behavior, which is difficult to predict from in vitro studies alone. In this study, we critically reviewed all research on different animal models investigating the osseointegration degree of new implant surfaces, reporting different species used in the osseointegration research over the last 30 years. Moreover, this is the first study to summarize reviews on the main animal models used in the translational research of osseointegration, including the advantages and limitations of each model and determining the ideal location for investigating osseointegration in small and large animal models. Overall, each model has advantages and disadvantages; hence, animal selection should be based on the cost of acquisition, animal care, acceptability to society, availability, tolerance to captivity, and housing convenience. Among small animal models, rabbits are an ideal model for biological observations around implants, and it is worth noting that osseointegration was discovered in the rabbit model and successfully applied to humans.

## 1. Introduction

Literature has revealed a high success rate (90–95%) of dental implants after five years of loading (including bone regeneration), and they are often used to rehabilitate partial or complete edentulous patients since dental implants are an effective treatment for tooth loss [1,2]. Rehabilitation of patients with tooth loss is challenging due to the reduced bone volume caused by tooth extraction, resulting in bone loss and alveolar ridge reduction, both vertically and horizontally. Such reduced bone volume and poor quality of the maxilla and mandible compromise clinical conditions, making implant rehabilitation unfeasible or impossible and increasing the implant failure rate [3].

Although titanium is extensively used to manufacture dental implants, it has insufficient osseointegration and osteoinductive features, especially in cases of inadequate or poor bone structure. Numerous modifications and improvements have occurred over time to enhance the performance of dental implants, especially for poor bone quality. In this regard, many initiatives in regenerative medicine are underway to develop new-generation biomaterials with high biocompatibility, aesthetic, and mechanical properties, as well as the ability to induce the adhesion and differentiation of osteoprogenitor cells, aiming to improve osseointegration and implant stability. As a result, new treatments of implant surfaces (e.g., sandblasting, acid etching, hydroxyapatite coating, and nano-coating with calcium, magnesium, strontium, zinc, and collagen type I) have been introduced to improve osseointegration by increasing bone–implant contact since they enhance the cell adhesion and proliferation of osteogenic cells [4,5]. For instance, graphene doped with poly-methyl methacrylate has recently been introduced due to its superior corrosion resistance and excellent aesthetic properties [6]. Given that many factors influence implant failure (e.g., implant insertion in the posterior and maxilla regions, untreated chronic periodontitis, heavy smoking, lack of initial implant stability, irradiation of the head and neck region, and poor quality of bone) [3,7], the research challenge is to find surfaces that are more osteoconductive to increase bone–implant contact and therefore reduce the implant failure rate. Hence, the biocompatibility and mechanical stability of such modifications in dental implant surfaces must be proved a priori in both in vitro and in vivo studies before clinical application in patients [8,9]. Although animal models may closely represent human physiological and mechanical conditions [10], they only approximate the clinical situation in humans since each animal model and anatomical area has distinct advantages and drawbacks when testing implant surfaces.

Since osseointegration is defined as the direct connection between living bone and a load-carrying endosseous implant at the microscopic level [11], animal models play a vital role in translational research investigating the osseointegration process. Although there is difficulty in translating findings from in vitro research to the clinical scenario, technological advancements are enabling us to create scenarios that mimic the dynamics of the human body using organ-on-a-chip technology [12]. Therefore, we critically reviewed all studies on various animal models studying the osseointegration degree of new implant surfaces, reporting different species used in the osseointegration research over the last 30 years. Moreover, this is the first study to summarize reviews on the main animal models used in the translational research of osseointegration, including the advantages and limitations of each model and determining the ideal location for investigating osseointegration in small and large animal models.

## 2. Use of Animal Models in Pre-Clinical Research

Despite significant advances in regenerative medicine and tissue engineering of implant dentistry, there was often debate concerning the use of animal models in translational research. In this light, ethical concerns have arisen, mainly in recent years, prompting the scientific community to evaluate and regulate the use of these models for human diseases, intending to reduce the potential risks and harms to the animals and the number of animals used for research [13]. Since 1986, the protection and well-being of animals in Europe have been covered by a broad spectrum of European Union regulations to enhance animal welfare and reinforce the “3Rs” concept (i.e., “Replace, Reduce, and Refine” the use of animals for research purposes) [14].

Although small animals (e.g., rodents) are valuable in studying human genetic diseases and several physiological processes, the research findings from these models are only sometimes translatable into clinical applications for humans [15,16]. Contrastingly, using large animal models (e.g., sheep, pigs, primates, and dogs) is still essential since they are clinically similar to humans in various aspects [17]. Therefore, many research-regulating organizations such as the European Medicines Agency (EMA), the United States Federal Food and Drug Administration (FDA), and the International Society for Stem Cell Research (ISSCR) are currently recommending to use large animal models whenever feasible to evaluate the efficacy, durability, dose-response, degradation, and safety of advanced therapeutic medicinal products (ATMPs) [18,19].

In 2010, the ARRIVE (Animal Research: Reporting In Vivo Experiments) guidelines were developed, including a checklist of recommendations for improving the reporting of animal research and enhancing the quality and reliability of published studies [20]. Moreover, such guidelines enable researchers to thoroughly investigate, evaluate, and reproduce the reported experiments and assist authors and journals in determining the minimal information required for reporting and describing in vivo experiments for publication [21]. However, the evidence reveals that many publications needed to include critical information, with significant scope requiring improvement in the reporting of research involving animals. As such, the ARRIVE 2.0 guidelines were published to emphasize that each study should define its specific hypotheses, methodologies, and statistical analysis plan before the investigation begins to minimize any risk of bias [20].

The animal model selection is paramount for obtaining credible results while adhering to the 3Rs principle. Hence, some factors should be considered, primarily the anatomical variances between species, as they may vary in consistency, composition, and biomechanical strength. Also, the animal’s species, sex, weight, and age should be considered, with an attempt to match the animal’s age with the age of the humans suffering from the investigated condition or pathology. As a result, these factors are critical since they could influence the reliability of the study’s findings (Table 1). Moreover, the ossification state is also essential because it influences bone response to biomaterials and assesses cellular response and bone quantity and quality around and in touch with the implant surface in osseointegration research. In this regard, international standards have concluded that the most suitable species for testing implantation materials in bone are dogs, sheep, goats, pigs, and rabbits. Therefore, we thoroughly analyzed the advantages and disadvantages of these species and the preclinical research that used these animals as models to evaluate osseointegration and related processes (Table 2).

## 3. Small Animal Models

### 3.1. Rat

In some cases, rats are a good choice among small animal models used in preclinical research, especially given the massive amount and quality of scientific study conducted over the last decades. Rodents (either mice or rats) are economical to house, breed, and acquire, and have around 4000 genes in common with the human genome (https://www.genome.gov/, Why mouse matters; accessed on 13 July 2022), making them an excellent choice for studying human hereditary diseases. Also, one of the critical advantages of employing rats is that they are widely accepted as laboratory animals by scientific societies, which facilitates their usage approval and minimizes ethical concerns about their use in preclinical investigations. Furthermore, they exhibit similar intestinal absorption profiles and transporter expression patterns in the small intestine, making them strong predictors of intestinal permeability [23].

However, their utility in bone defects and osseointegration research remains limited to specific experimental strategies due to anatomical and biomechanical differences from the human skeleton and distinct metabolism and enzyme patterns that could complicate the analysis of the results. As a result, rat models have mainly been used to assess the toxicity and biocompatibility of various dental implant materials. For instance, Scarano et al. employed this model to evaluate the biocompatibility of a material commonly used in coating surgical instruments (i.e., titanium nitride [TiN]) in dental implants They were able to demonstrate with minimal and sufficient number of animals that TiN coating for dental implants is an optimal solution for achieving good biocompatibility without effects on the peri-implant bone formation and the surface roughness values [24].

Such animal models are used for evaluating the healing of bone defects (typically via calvaria); however, both femoral and tibial bone can be used to research implant surfaces, whereas calvaria is utilized to assess bone grafts and regeneration. As such, it is evident that the implants should be miniscule in size due to the risk of femur or tibia fracture during implant screwing. In previous research, we employed implants with 2 mm diameter and 2 mm length, since preparing the implant site to evaluate the influence of insertion torque on healing around implants is impossible [24]. However, postoperative accidents and complications are infrequent since these animals do not make brisk movements that could fracture the femur after implant placement. The tibial and femoral bone structures have a large medullary cavity and cortical bone thickness of 0.5–0.7 mm; hence, implants with 1.5–2 mm diameter and 2 mm length can be placed there since such anatomical structures allow for rapid assessment of cellular responses to the implant surface. As such, rats are pharmacologically and economically manageable animals because bone healing around the dental implant is represented by inflammatory cells after five days, with osteoblastic differentiation peaking at 20 days; thus, we get a well-integrated implant at 30 days.

**Figure 1:**
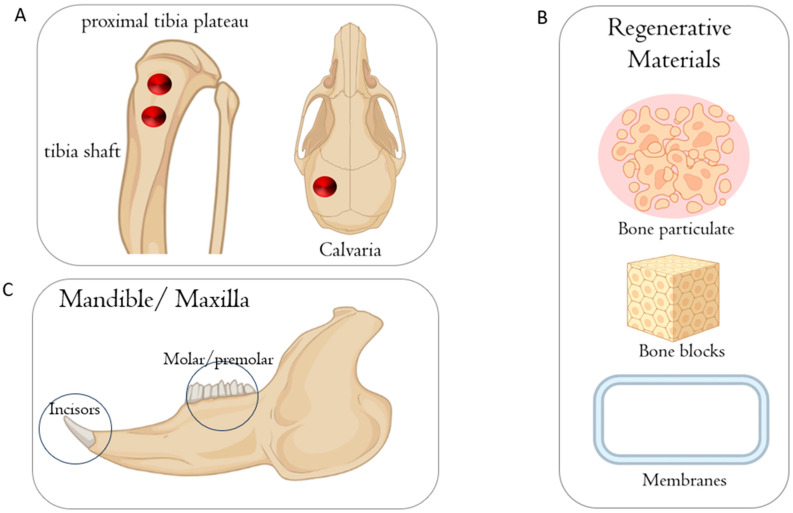
Schematic illustration of the anatomical sites used on rats to evaluate biomaterials during bone healing. (**A**) Bone defect in the tibia and calvaria. (**B**) Different biomaterials used for histological evaluation. (**C**) Edentulous space, incisor, and premolar that can be used to insert mini-implants or post-extractive implant. This figure was created using Biorender.com.

**Figure 2:**
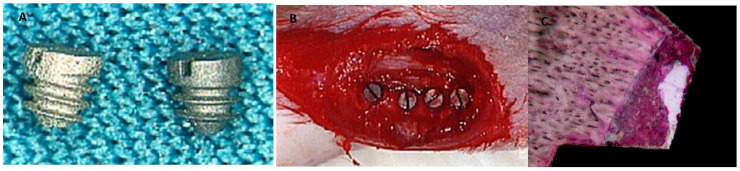
(**A**,**B**) Mini-implants positioned in tibia. (**C**) Histological aspect of mini-implant after bone healing [24].

Nevertheless, there is considerable disagreement about the similarity of rat bone remodeling compared to humans [25,26], as some perspectives assume that rat bones differ from human bones due to their ash and collagen contents; hence, rodents are not appropriate models for studying bone biology [27,28]. Overall, recent research has shown that there is no evidence of the superiority of pig models over rodent models in representing human bone biology [29]. Other viewpoints hold that rats have the same growth factors, cytokines, and chemokines that regulate the bone remodeling process in humans and that these similarities justify using rats in osseointegration research [30,31]. For instance, recent studies used rats to evaluate the effect of smoke exposure on the bone metabolism around implants coated with nano-hydroxyapatite [32] and the influence of nano-roughened surfaces on the osseointegration strength [33] (Figure 1). Also, this animal model was used to evaluate the collagen fiber formation around an implant during the osseointegration process [34] and compare the histological levels of osseointegration of titanium vs. zirconia dental implants [35]. The operative protocol is general anesthesia with 50 mg/1 kg of ketamine hydrochloride and 10 mg/1 kg of xylazine hydrochloride, followed by trichotomy and antisepsis with a 1% povidone–iodine solution. In the mesial area, a skin incision is then made parallel to the long axis of the tibia with a type 15 scalpel blade to expose the tibia surface. It is an animal model widely used in research (Figure 2).

### 3.2. Rabbit

**Figure 3:**
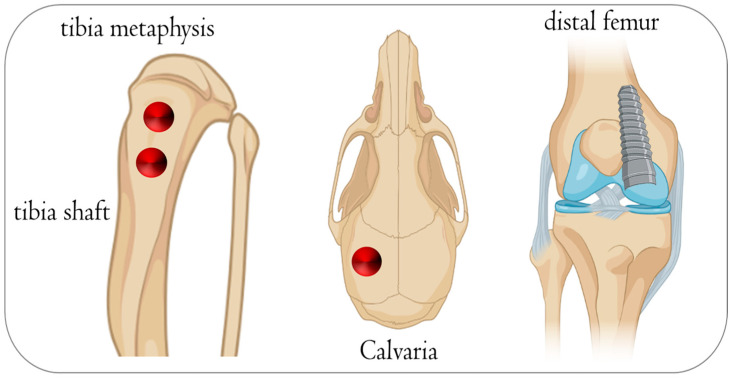
Schematic illustration of the anatomical sites used on rabbits to evaluate biomaterials during bone healing. Bone defect to simulate post-extraction sites. This figure was created using Biorender.com.

**Figure 4:**
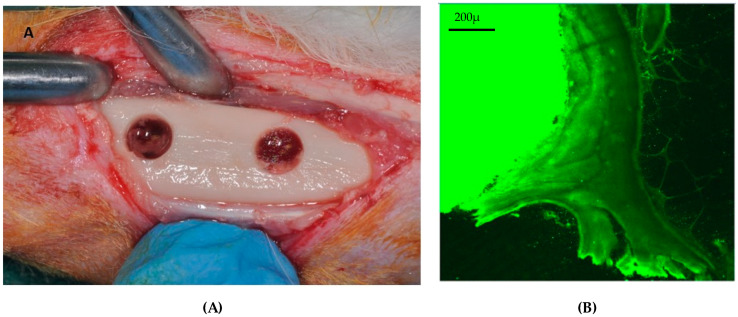
(**A**) Bone defect in the tibia. (**B**) Histological specimen of dental implant positioned in the tibia and observed under a fluorescence microscope [43].

Rabbits are small mammals commonly used in preclinical studies before testing implant materials in larger animal models, primarily due to their widespread availability, low economic cost, easy handling, and non-aggressive nature. Moreover, rabbits possess short vital cycles of gestation, lactation, and puberty, giving them an essential role in translation research. However, the macrostructure and microstructure of rabbit bone are not similar to human bone, since rabbits undergo faster skeletal alterations and bone turnover than humans [36,37]. Because bone fractures and bone defects heal faster in rabbits than in humans, dental implants placed in rabbits may only take 5–6 weeks to osseointegrate [38], whereas it takes 3–4 months in humans [39]. Still, many implant investigations have been staged in the rabbit tibia, primarily since fundamental osseointegration discoveries were established in the rabbit model [39] (Figure 3). Brånemark studied intravital microscopy and noticed that titanium camerines were undergoing a process of bone integration [40] (Figure 4).

**Figure 5:**
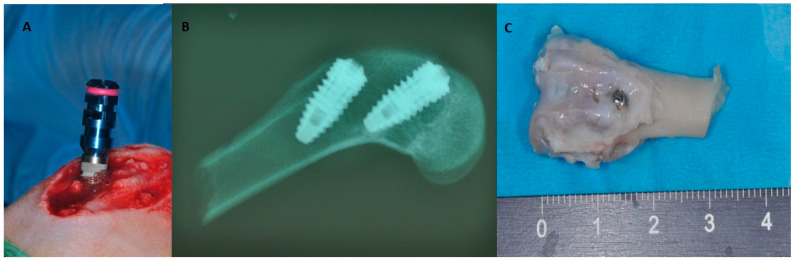
(**A**) Dental implant with standard dimension (4 × 13 mm) during placement in the knee joint. (**B**) Radiographic assessment of two dental implants placed in the knee joint. (**C**) Block section of knee joint before histological analysis [44].

However, utilizing rabbit models in implant research has several limits because rabbit bones are weak and lightweight [41,42], and their mandible size does not allow for a standard-sized dental implant placement. Preclinical tests are typically carried out on the mid-femur, tibia, or knee joint despite the tiny diameter of 0.5 cm, which makes implant placement challenging [37,43]. Also, a standard-sized dental implant with a 4 mm diameter and 13 mm length could be placed in the femoral epitasis and knee joint (Figure 5). As a result, the mandible has been used only infrequently for mini-implant placement in the anterior edentulous space distal to the incisor tooth. This animal model is also used for the evaluation of biomaterials in sinus lift prior to dental implant placement.

Nano- and micro-topography, along with the physicochemical composition, are two characteristics of the implant surface that influence the osseointegration outcomes and allow for biological and morphological compatibility. For instance, Scarano et al. compared two implant types (a chemically activated calcium-modified surface (CA) implant and an SLActive surface implant) inserted into the articular femoral knee joint of rabbits to study the differences between implants with different shapes and macro-design. They evaluated the proportion of mineralized tissues at the bone–implant interface using bone intact contact (BIC), which resulted in equal values for both implant types and excellent osteoconductive response [45]. Further research was conducted on such models by creating a critical defect (6 mm diameter) in the rabbit tibia to evaluate the influence of autologous platelet gel (APG) derived from platelet-rich plasma (PRP) on bone healing [43], as it has been extensively studied as a periodontal therapy and in the healing process of tissues, with successful results [46]. Given that PRP has been proposed as support to minimize the autologous bone required during regenerative surgery, Scarano et al. aimed to further investigate the bone regeneration enhanced by APG using an optimal animal model such as the rabbit. They found a higher quantity of lamellar and woven bone in the injured zone compared to the control and a difference in the percentage of new bone formation and marrow space, with an increased bone formation in the APG group when compared to the woven bone and an increased formation of mature (lamellar) bone restricted to the cortical bone [43].

Furthermore, numerous fundamental investigations in the cuniculus model have shed light on the involvement of micro- and macro-geometry in bone healing and neoangiogenesis [47,48]. The beneficial outcomes achieved regarding implant materials, biocompatibility, osteoconductivity, and BIC (among others) significantly support the rabbit’s crucial role as an animal model for studying essential elements in dentistry. The operating protocol includes intramuscular injections of diazepam (1.5 mg/kg b.wt.) and fluanizone (0.7 mg/kg b.wt.), followed by local anesthesia with 1 mL of 2% articaine/adrenalin solution. To accomplish skin asepsis, the skin around the anatomical area’s must be shaved and disinfected with a 2% chlorhexidine gluconate antiseptic solution. Afterward, a skin cut with a periosteal flap must be used to expose the tibia, femur, or femur knee (articular surface).

## 4. Large Animal Models

### 4.1. Sheep

**Figure 6:**
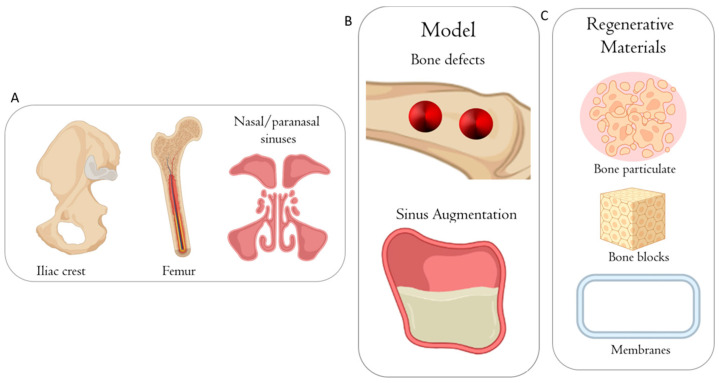
Schematic illustration of the anatomical sites used on sheep to evaluate biomaterials during bone healing. (**A**) Bone defects in tibia, femur, and sinus lifting. (**B**) Different biomaterials used for bone defect treatments. (**C**) Biomaterials in particles, blocks, or membranes that can be used to treat bone defects. This figure was created using Biorender.com.

**Figure 7:**
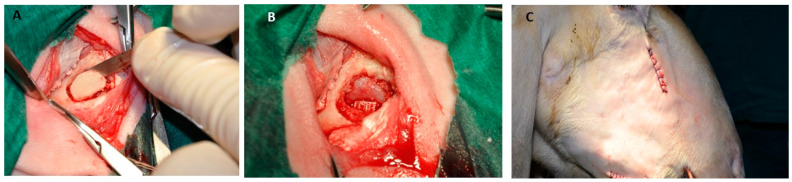
(**A**) Osteotomy for sinus lifting. (**B**) Sinus membrane lifting. (**C**) Extra-oral skin suture [59].

Sheep are considered good large models for translational research due to their low housing cost, docility, wide availability, and easy handling. Also, their hormone profile is similar to that of women, giving them a potential role in the research of human pathologies associated with osteoporosis and hormone imbalances [49]. As a result, ovine models are employed with multiple applications ranging from studying reproductive disorders to cardiopathologies, orthopedics, and even dentistry. The key reason is their anatomical, biomechanical, and physiological similarities to humans since their bone composition, joint structure, architecture, and weight are comparable to ours [50] (Figure 6 and Figure 7).

**Figure 8:**
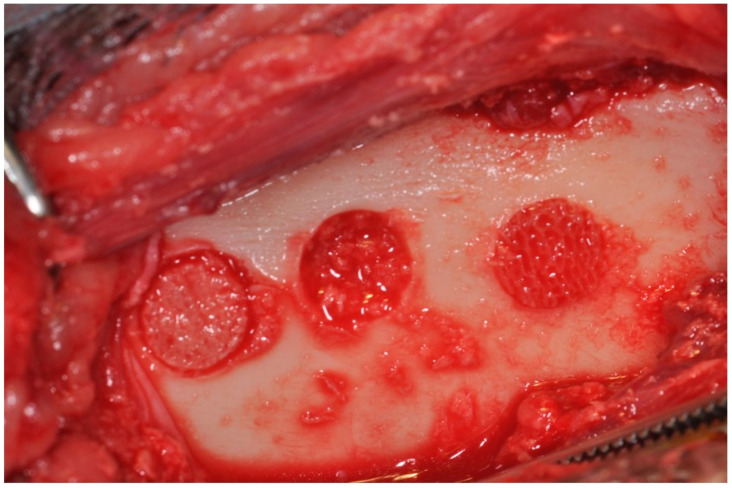
Bone defects in the tibia are used to evaluate different bone grafts [60].

The tibia, femur, and iliac crest are typically employed for osseointegration examination, whereas the sinus is used to evaluate the various potential regenerating bone substitute graft biomaterials (Figure 6, Figure 7 and Figure 8). Given that mature sheep have a bodyweight equivalent to adult humans and long bone dimensions, they are utilized to evaluate the performance of implant surfaces. Moreover, their cortical and trabecular bones have a similar structure to human bone, mimicking human bone physiology and anatomy in adult animals [51]. Furthermore, sheep bones have similar mineral compositions to humans [52], and both metabolic and bone remodeling rates are equivalent [53], with comparable bone blood supply and bone healing capacity [54]. As a result, sheep are an excellent model for researching cortical and medullary bone healing around implants. Thus, various dentistry investigations have been conducted using sheep models [55]. For instance, sheep were utilized to study the in vivo tissue response and gap-healing patterns of four differently treated dental implants by placing them into pre-created iliac crest lesions. Such preclinical studies were beneficial in shedding light on two materials (particulate porcine bone mix and porcine corticocancellous collagenated pre-hydrated bone mix) that could stimulate bone regeneration when utilized as scaffolds, giving excellent biocompatibility [56]. Also, the ilium crest was utilized to evaluate the osseointegration of dental implant surfaces [57], while the medial femoral condyles were employed to assess bone repair around biomaterials Furthermore, sheep have been used to evaluate the biomaterials used in the sinus lift procedure [58].

As always, the ovine models have some limitations that should be considered in the study design. First, sheep have higher trabecular bone density than humans. Second, they are seasonal breeders with varying estrogen levels throughout the year [50,61]. The operative protocol is anesthesia with intravenous injection of 0.5–1.5 mg thiopental atrium, followed by endotracheal intubation with a 9 mm tube. For maintenance of the anesthesia, a combination of anesthesia gas (1.8–2.0% isoflurane, pure oxygen, 20–30% nitrous oxide) and fentanyl dihydrogen citrate in a dosage of 0.2 mg per kg body mass as intravenously bolus injections for analgesia must be delivered.

### 4.2. Minipig

Minnesota miniature pigs have been frequently employed in preclinical investigations for economic, ethical, and scientific reasons since they were first developed in 1949 at the Hormel Institute in the United States. The close anatomical similarity of minipigs to humans in terms of development, physiology, pathophysiology, and disease occurrence is well-known, and they have human-like maxillar and mandibular dimensions and alveolar bone remodeling, making them a valuable animal model for evaluating osseointegration and studying oral and maxillofacial [62], sensorineural [63], toxicological [64], and other diseases [62]. In anatomy, development, physiology, pathophysiology, and disease incidence, the oral maxillofacial region of miniature pigs resembles that of humans. Also, their bone mineral density and architecture are similarly histologically and radiographically comparable to humans [65]. The bone-regeneration capacity of minipigs is nearly identical to ours, with humans recovering 1.0–1.5 mm per day and minipigs recovering 1.2–1.5 mm per day [66]. As a result, such a model has been widely used to evaluate the effect of implant geometry and surfaces on new crestal bone formation and bone–implant contact [67], as well as the new implant surfaces and immediate loading [68,69,70].

**Figure 9:**
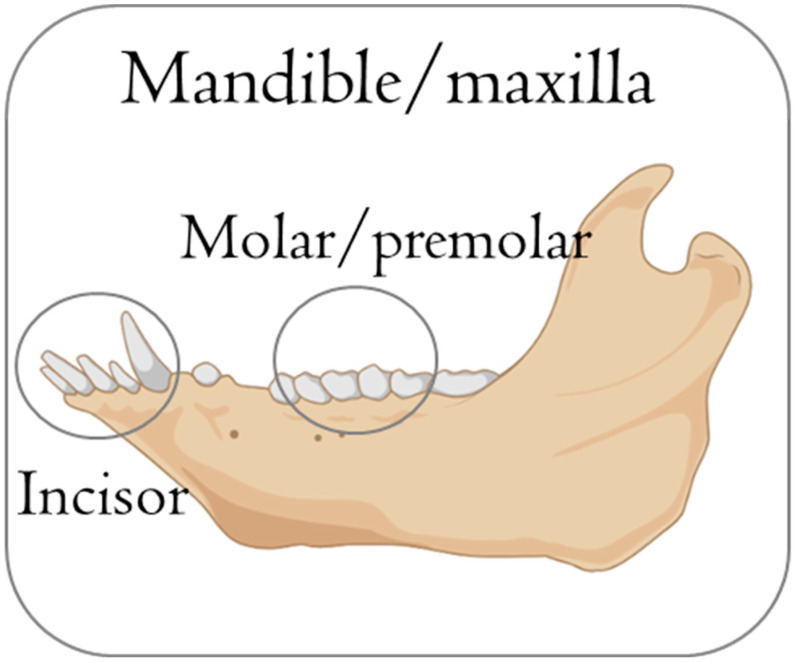
Schematic illustration of anatomical sites used in minipigs to evaluate biomaterials. Different biomaterials used for bone defect treatments. Anatomy of teeth shows their complexity; it is challenging to perform extractions without fracturing the roots. This figure was created using Biorender.com.

**Figure 10:**
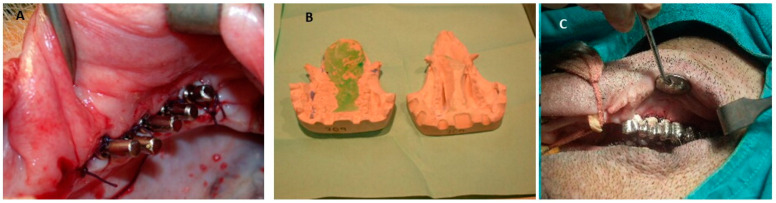
(**A**) Dental implants placed in the maxilla. (**B**) Plaster model after impressions for denture construction. (**C**) Metal prosthesis placement [73].

Furthermore, minipigs have been recommended as an alternative to canine models, which are fraught with ethical and controversial concerns. One of the most significant advantages of using the minipig model is its deciduous, mixed, and permanent dentitions, with the permanent molar being the first tooth to erupt, and an extended mixed dentition period [65]. However, the morphology, number of teeth, and tooth eruption differ since minipigs have six maxillary/mandibular incisors instead of four and eight maxillary/mandibular premolars instead of four [65]. Also, a radiographic and micro-CT study found that minipigs have thinner roots than humans, and their molars have five roots, whereas premolars have three to four roots [71]. As such, the implants were placed in the mandible in the tibia, femur, and iliac crest in this animal model; however, many researchers often choose extra-oral sites since implants positioned in the alveolar area require preventive tooth extraction, which lengthens research time and expenses. Tooth extraction in minipigs requires extreme caution since their roots are highly divergent and usually break off (Figure 9 and Figure 10).

Pilawski et al. revealed that minipigs have collagen organization, osteocyte density, alveolar bone remodeling, and mineral apposition similar to humans, as well as comparable bone volume, architecture, and mineral density [29]. Given these physiological similarities to humans, minipigs have been used to evaluate several diseases induced in pig models; hence, they are a valid model for xenotransplantation research and evaluating many human diseases (e.g., orthopedic, cardiovascular, and dermatological backgrounds) [29,72]. Generally, large animal models outperform small animal models like rodents in various aspects of investigating human diseases and preclinical research. The operative protocol is general anesthesia with Zoletil 100 (tiletamine hydrochloride + Zolazepam hydrochloride) induction at 6 mg/kg IM dosage and maintenance with isoflurane at 2/2.5% in Oxygen.

### 4.3. Pig

Pigs have various advantages over other animals due to their anatomical, physiological, metabolic, and genetic similarities with humans, including skin structure, cardiovascular system, urinary system, and immune system [72]. Also, pigs are more similar in weight and size to humans, which is why this animal model has been frequently used in biomedical research, especially over the last 30 years, to investigate a range of human diseases. Generally, domestic pigs are frequently used as animal models due to their rates of bone regeneration (1.2–1.5 m/day) that are similar to those of humans (1.0–1.5 m/day) [50]. In implantology, both pigs and minipigs have similar anatomic characteristics to humans, allowing for using such species in evaluating dental implants that could subsequently be extrapolated to humans. Thus, the maxillary and mandibular bones of minipigs and pigs are among the most commonly used models for dental implant studies [74].

However, pig molar extraction is challenging due to their enormous size and root shape, and the pig’s bulk (150–200 kg) against the minipig’s (20–40 kg) makes it more difficult to handle during the operation phases [75]. Hence, the primary drawback of this procedure is the necessity of tooth extraction prior to implant placement, as well as a higher complication rate due to contamination of intraoral contents as they begin to eat and function normally again [76]. Despite pigs having a bone structure and physiology similar to minipigs, this animal model has been abandoned in implantology research due to its more difficult management issues. Therefore, the recent literature shows considerable implant research conducted solely on minipigs, with pigs utilized to investigate immediate loading and bone remodeling processes surrounding implants loaded with metal and acrylic resin prosthetic restorations, resulting in no differences in the peri-implant bone response between two restorations. Based on our observations, maxillary mandibular bone of pigs has a higher percentage of fat tissue than other animals. Another issue this animal model raises is the difficulty of extracting the roots without leaving residue in the alveolar socket, which could lead to infection. As a result, many researchers choose to evaluate the implant surface in another anatomical location, such as the tibia or femur [77]. The anesthesia protocol includes an intramuscular injection of sulfadiazine, followed by an intramuscular injection of 0.07 mL/kg of a mixture of 12.5 mg tiletamine, and 12.5% zolazepam, 12.5 mg xylazine, 12.5 mg ketamine, and 2.5 mg, while artificial respiration with 0.3–1% isoflurane is used for maintaining anesthesia.

### 4.4. Dogs

For centuries, dogs have been used in drug testing and surgical research because dog diseases are similar to human diseases; thus, they have been used as a model for many human conditions such as diabetes mellitus, cardiovascular research, open-heart surgery, ulcerative colitis, pharmacology and toxicology, and organ transplantation [66]. Dog teeth differ numerically and morphologically from human teeth; however, dogs have been considered the traditional model for studying periodontitis for years since most canines can acquire periodontal diseases with a conventional transition process from gingivitis to periodontitis, similar to humans [78]. Also, the evaluation of bone–implant interface and bone healing in dog models are often a common aspect of the final preclinical research stages. The bone composition and density of dogs are comparable to those of of humans [27], and the capacity for bone remodeling in dogs and humans is nearly identical: 05–6.4% in dogs and 3–4% in humans [79,80].

**Figure 11:**
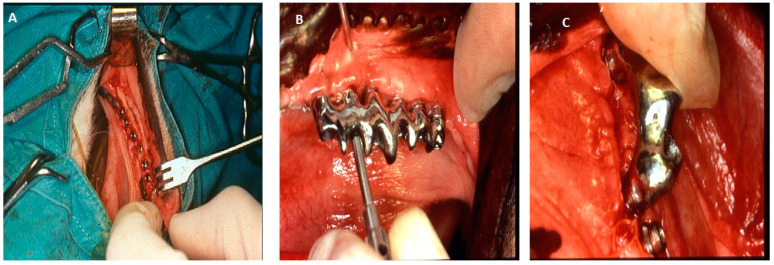
(**A**) Implants placed in mandible. (**B**) and (**C**) Prosthesis placement [81].

Most research is usually undertaken in the posterior mandible, since the mandibular alveolar process is thicker, whereas the maxillary alveolar process is extremely thin and porous [79]. Also, the maxilla of dogs has relatively thin bones, and their nasal cavities are very close to the alveolar process. The tibia and femur are rarely employed because their remodeling capacity is less than that of the maxilla and mandible, which are 3 and 6 times greater, respectively. As such, experiments on dogs have included the investigation of crestal bone behavior around dental implants placed with varying inter-implant distances, aiming to evaluate the potential influence of these distances on lateral bone loss and crestal bone resorption [81] (Figure 11). These findings, along with those of Tarnow et al., emphasized the importance of using implants with smaller diameters to reduce inter-implant bone loss, as increasing crestal bone loss increases the distance between the base of the contact point of the adjacent crowns and the crest of the bone [81,82]. Another study found that the position of the micro-gap influences bone crestal level changes in the loaded implant [81]. Given that implants were mostly placed in dogs’ mandibles, many studies have been conducted to evaluate the effects of implant superficies, crestal remodeling, post-extraction alveolus, and post-extraction implants [83,84]. However, translational research employing the canine model is always controversial due to their role as companion animals and their consideration as family members.

## 5. Discussion

Many researchers have focused on the physical and chemical modification of treated implant surfaces to increase cellular activities and promote implant integration with surrounding bone. Hence, researchers have been modifying and developing new dental implant surfaces for many years to optimize the interaction between the body and the implant. The high percentage of bone–implant contact fosters the clinical success of titanium dental implants for rehabilitating partial or total edentulous patients. Since it is essential to determine whether the new implant surface is biocompatible and mechanically stable before the clinical application, it must undergo rigorous investigations in vitro and subsequently in vivo. As such, in vitro testing is employed primarily as a first-stage test for cytotoxicity, genotoxicity, cell differentiation, and proliferation to avoid the unnecessary use of animals. Similarly, before clinical application on humans, an animal model is required to evaluate the tissue response and mechanical function of the new implant material. Dental implants can be investigated in healthy or osteopenic bone under loading or unloading circumstances for potentially extended periods. It should be noted that animal models can resemble the human clinical physiological and mechanical state, but this is simply an approximation, and each animal model has distinct advantages and limitations [85]. Therefore, we reviewed the literature that used animal models to evaluate bone–implant interactions over the last three decades.

Overall, new dental implants necessitate in vivo testing to verify their biological response as defined by micro- and macro-morphology. Another regulation requirement (ISO 7405:2018) is that implants be examined in their final shape and size before being used in humans [86]. For instance, the biological response evaluation requires using a rabbit model; thus, implants should be placed in the tibial and femoral diaphyseal bone without exceeding 3.75 mm in diameter and 8 mm in length. Although such an animal model heals faster than humans (particularly in medullar space), this difference in healing speed is advantageous since it amplifies the biological reaction, enabling the results to be better interpreted. On the other hand, evaluating the loading biological effects and other clinical situations necessitates using large animal models (e.g., sheep, minipig, or dog) since they allow for the placement of implants with 3.75–4.5 mm in diameter and 8–12 mm in length. Also, using the posterior mandible as an anatomical area is preferable when choosing large animals. However, sheep chew differently than humans and suffer from periodontal disease; thus, they cannot be employed in implant function research, and their applications are limited to sinus lift or implant placement in long bones or iliac crest [56]. In addition, the difficulty in obtaining ethical permission, which usually recommends against using dog models, is a crucial cost consideration when evaluating the dog model.

Accordingly, researchers should look for alternative animal models, and their decision should be based on the cost of acquisition, animal care, acceptability to society, availability, tolerance to captivity, and convenience of housing. In this regard, small animal models (e.g., mouse, rat, and rabbit) have helped study a limited number of events; in contrast, large animal models provide necessary insights to elucidate the complex events within the physiological oral environment. For instance, rabbits are suitable animal models for estimating the influence of implant surface modifications on osteogenesis and bone–implant interaction responses, since they are inexpensive to acquire and care for, have societal acceptability, availability, tolerance to captivity, and ease of housing. Thus, large animals are only justified in research investigating the effect of loading on soft and hard tissues, and rabbit models remain suitable for evaluating the influence of macro- and micro-morphology on bone healing around the dental implant. However, implants can only be placed on the tibia or femur of rabbits; hence, there is a significant drawback for dental implant placement due to extensive bone marrow space under the cortical bones, which is attributed to the different comparison and micro-architecture of femur and tibia with maxillofacial bones due to endochondral ossification. In contrast, minipigs represent a pivotal model for preclinical evaluation of dental implants, but remodeling rates in their long bones are 3–6 times lower than in the maxilla and mandible [79]. Also, dog and minipig models have substantial disadvantages for an intraoral approach since tooth extraction is necessary before dental implant placement; thus, there is an increased complication rate (exposure of grafts and implants discovered) due to contamination of intraoral contents [76]. Furthermore, the canine model has additional drawbacks because the breeds employed are of varying sizes and are frequently opposed by society and animal welfare organizations. Non-human primate models were considered very similar to humans and have been used in the past for the evaluation of immediate loading, occlusion, and preangled abutments [87]. Today, for ethical reasons, as well as for cost and housing difficulties, non-human primates have almost completely stopped being used in dentistry.

## 6. Conclusions

In conclusion, no animal model can completely simulate the loading circumstances of human dental implants; however, among small species, the rabbit represents an ideal model for biological observations around implants without the influence of loading. It is also worth noting that osseointegration was discovered in the rabbit model and successfully applied to humans. Dogs and sheep represent good animal models for evaluating the loading of standard-size dental implants, while rats are a good low-cost animal model to study the influence of surfaces on bone healing, although it requires the use of very small dental implants.

## Figures and Tables

**Table 1:** Comparison of the main physiological, anatomical, and biomechanical features of the species commonly used in osseointegration research [13,22]. Table modified from Ribitsch et al., Frontiers in Bioengineering and Biotechnology [13].

Animal Features	Species					
	Rat	Rabbit	Dog	Minipig	Pig	Sheep
Body temperature (°C)	37.5–39.5	38–39.5	38–39	38.3–38.8	38–40	38.5–39.5
Heart rate (bpm)	250–450	150–300	60–160	68–72	90–100	60–80
Respiration rate (pm)	70–120	35–100	15–30	14–18	10–20	16–30
Maximum weight (kg) *	0.2–0.5	5–6	1–30	20–40 *	150–400	40–70
Mean life span (years) *	2.5–3.5	5–15	10–15	15–18	15–20	10–12

* Depending on the breed.

**Table 2:** Advantages and disadvantages of the species most frequently used in osseointegration research.

Species	Advantages	Disadvantages
Rat	Easy handling Low housing and breeding costs Widely available Short life span Short vital cycles Laboratory animal consciousness Model of diseases Easy genetic manipulation	Small size Huge differences compared to the human skeleton Limited surgery
Rabbit	Easy handling Low housing and breeding costs Widely available Docile Relatively short life span * Short vital cycles	Small size Macro and microstructures differ from human Limited surgery
Dog	Bone properties similar to human	Major ethical concerns (companion animals) Long life span Elevated costs Special facilities required
Minipig	Bone properties similar to human	
Pig	Size and weight near to human Good for surgery Bone properties similar to human Genetic tools available	Long life span Relatively elevated housing and breeding costs
Sheep	Size and weight near human Relatively low housing and breeding costs Widely available Hormone profile similar to women Good for surgery Bone properties similar to human	Long life span Seasonal breeders Higher trabecular bone densities in some locations

* Depending on the breed.
